# Sensing behavior of CdS-TiO_2_ thick films for the detection of hydrocarbons

**DOI:** 10.1039/d4ra05824k

**Published:** 2024-12-03

**Authors:** Ankit Kumar Vishwakarma, Ajaya Kumar Sharma, Arpit Verma, B. C. Yadav, Lallan Yadava

**Affiliations:** a Department of Physics, Deen Dayal Upadhyaya Gorakhpur University Gorakhpur U.P. 273009 India kv.ankit92@gmail.com nisaly06@rediffmail.com; b Nanomaterials and Sensors Research Laboratory, Department of Physics, Babasaheb Bhimrao Ambedkar University Lucknow-226025 U.P. India

## Abstract

In this article, the sensing behaviors of undoped titanium dioxide (TiO_2_) and CdS-doped TiO_2_ (CdS-TiO_2_) thick films are discussed. Sensing pastes of 2 wt% CdS-TiO_2_ and undoped TiO_2_ were prepared in the laboratory and used to fabricate thick film gas sensors on an alumina substrate. The crystal structures of TiO_2_ and CdS-TiO_2_ samples were characterized by XRD and atomic force microscopy (AFM). The results indicated that the grain size and RMS roughness parameter were reduced by adding CdS contents. The sensing behaviors of the fabricated devices were studied at varying concentrations (0–5000 ppm) of different hydrocarbon gases, such as LPG, methanol, ethanol, toluene, and benzene, in ambient air at 300 K. The effect of humidity levels on the sensing properties of the sensors was also investigated. The sensor response value of CdS-TiO_2_ for benzene was found to be 2.25 times higher than that of TiO_2_-based sensing devices. Thus, CdS doping significantly enhanced the response and recovery times of the sensor. The TiO_2_ film exhibited response and recovery times of 65 s and 180 s, respectively. In contrast, when doped with CdS, the response times were reduced to 15 s and 103 s, respectively, when exposed to benzene at a concentration of 5000 ppm at 300 K. The sensing mechanism has been discussed and the experimental results were validated using a model based on the Frenkel–Poole theory of electronic emission and catalytic oxidation. The obtained results demonstrate that TiO_2_ structures doped with low concentrations of CdS exhibit superior sensitivity and selectivity to benzene gas under low humidity levels at room temperature (300 K).

## Abstract 2

Undoped titanium dioxide (TiO_2_) and CdS-doped TiO_2_ (CdS-TiO_2_) thick films were used to fabricate thick film gas sensors on an alumina substrate.

## Graphical abstract

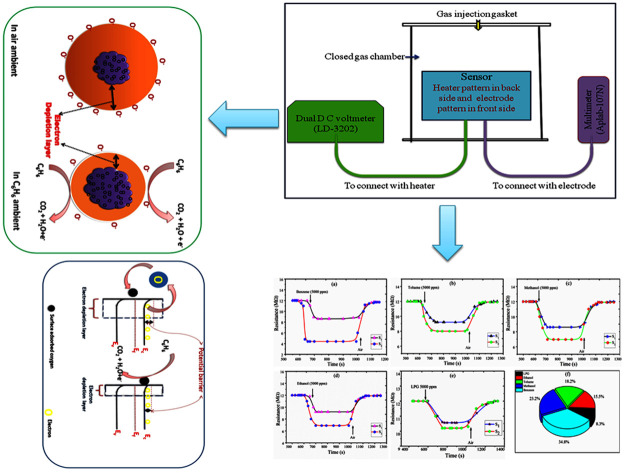

## 1. Introduction

In recent years, the necessity for the development of sensitive and stable hydrocarbon gas sensors has increased significantly. Detection of hydrocarbons and volatile organic compounds (VOCs), such as benzene, and toluene gases, is a subject of growing importance in domestic and industrial purposes. n-Type semiconductor oxides, such as TiO_2_, SnO_2_, ZnO, and WO_3_, have been extensively investigated as chemo-resistive gas sensors.^1,2^ Among these, TiO_2_ is a promising material, which is frequently used in industry, research, and environmental monitoring. Due to its semiconducting nature and better chemical properties, several researchers have used TiO_2_ and its dopants, such as Pd,^3^ Pt,^4^ Al,^5^ Nb,^6^ (Er^+3^),^7^ and Cr,^8^ to detect a wide variety of gaseous species, including O_2_,^9^ H_2_,^10^ CO,^11^ NO_*x*_^12^ LPG and benzene.^13^ The combination of CdS and TiO_2_ has been the most useful in detecting toxic, inflammable, and hazardous gases and is used in solar cells and photocatalytic applications.^14^ A. L. Micheli developed the first TiO_2_ gas sensor which was primarily used to detect stoichiometric air-to-fuel ratios.^15^ According to a research by S. Shao *et al.*, hierarchical nanospheres made of rutile and anatase phases show superior gas sensing capacities at ambient temperature, in either an n-type or p-type fashion. The researchers found that the addition of Pt decorations had a significant impact on limiting the grain size, increasing the surface area, and regulating the particle size. Particularly, it was demonstrated that altering particle size and increasing surface area increased sensitivity to ethanol (an n-type property) and benzene (a p-type property), respectively, at ambient temperature.^16^ Y. Wang *et al.* studied the synthesis and gas sensing properties of La and V co-doped TiO_2_ thick films.^17^ They observed that conductance increased with V and La doping and could inhibit the transformation from the anatase to rutile phase. The gas sensor based on La and V doping thick films showed excellent response to methylbenzene. D. Zhang *et al.* reported the fabrication of a Pd-decorated TiO_2_/MoS_2_ ternary nanocomposite for enhanced benzene gas sensing performance at room temperature, and they found that the fabricated sensor exhibited a high response, fast response–recovery time, repeatability, and good selectivity toward benzene.^18^ H. Bian *et al.* investigated the characterization and gas-sensing properties of electrospun TiO_2_ nanorods, concentrating on their acetone sensitivity. They found that while detecting acetone gas, these TiO_2_ nanorods exhibited quick reaction times, maximum sensitivity, increased selectivity, and long-term stability. These results led the scientists to the conclusion that TiO_2_ has a lot of potential as a material for the high-temperature detection of acetone.^19^

Benzene is widely used in chemical processes to produce products such as plastics, pesticides, pharmaceuticals, and other chemicals.^20^ Benzene is a carcinogen having hazardous properties, which causes health issues, particularly in urban areas. Therefore, it is imperative to monitor benzene for health and safety. In the present research work, for the detection of benzene, a sensing device based on the CdS-TiO_2_ structure has been investigated. The influence of CdS content on the microstructural properties and its sensing capability for various gases, including toluene, benzene, ethanol, methanol, and LPG, at room temperature have been investigated. The effect of humidity levels is also studied. A smaller CdS content (2 wt%) remarkably enhanced the sensitivity of TiO_2_ to benzene gas in comparison to other test gases. The sensing mechanism and experimental results were justified by mechanistic and experimental results.

## 2. Experimental details

### 2.1. Fabrication of thick film sensor and sensing setup

**Fig. 1:**
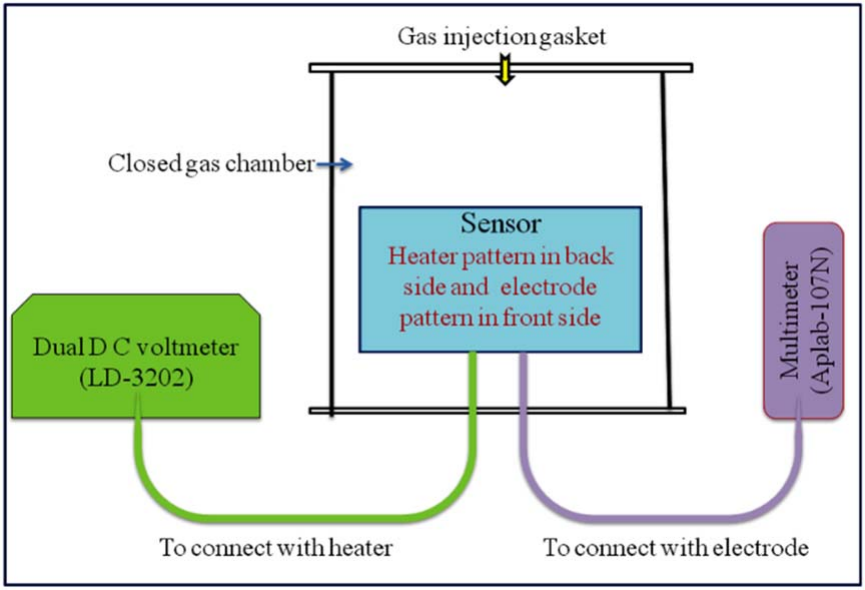
Block diagram of the measurement setup.

Undoped TiO_2_ (S_1_) and 2 wt% CdS-doped TiO_2_ (S_2_) thick films having dimension of 8 mm × 8 mm were fabricated on alumina substrate of dimension 25 mm × 25 mm. The finger electrode pattern was printed using silver conductor paste (paste FD6176) on the front side, and the heater electrode pattern was printed using ruthenium oxide-based resistor paste (paste NTC 2413 ESL) on the backside of the substrate. The printed thick films were annealed at 250 °C for 90 min to ensure good adherence of the sensing layer on the substrate. For the measurement of the responses of the sensors (S_1_, S_2_) we designed a locally made glass chamber of volume 2047 mL in which provisions were made for the injection of gas and electrical connections. The electrical resistance of the sensors was measured with varying concentrations (0–5000 ppm) of different test gases, including benzene, toluene, methanol, ethanol, and LPG, at a temperature of 300 K using the experimental setup shown in Fig. 1.^21^

### 2.2. Characterization

The microstructural properties of the fabricated thick film sensors S_1_ and S_2_ were studied using X-ray diffraction (XRD) and atomic force microscopy (AFM). XRD pattern was obtained by a D8-advanced equipped with Cu Kα_1_ radiation of wavelength 0.15406 nm, while surface morphology was investigated using AFM. The AFM image was recorded with the digital instrument, Nanoscope-IV, with a Si_3_N_4_ 100 μm cantilever and 0.58 N m^−1^ force constants in contact mode.

## 3. Results and discussion

### 3.1 Microstructural properties

**Fig. 2:**
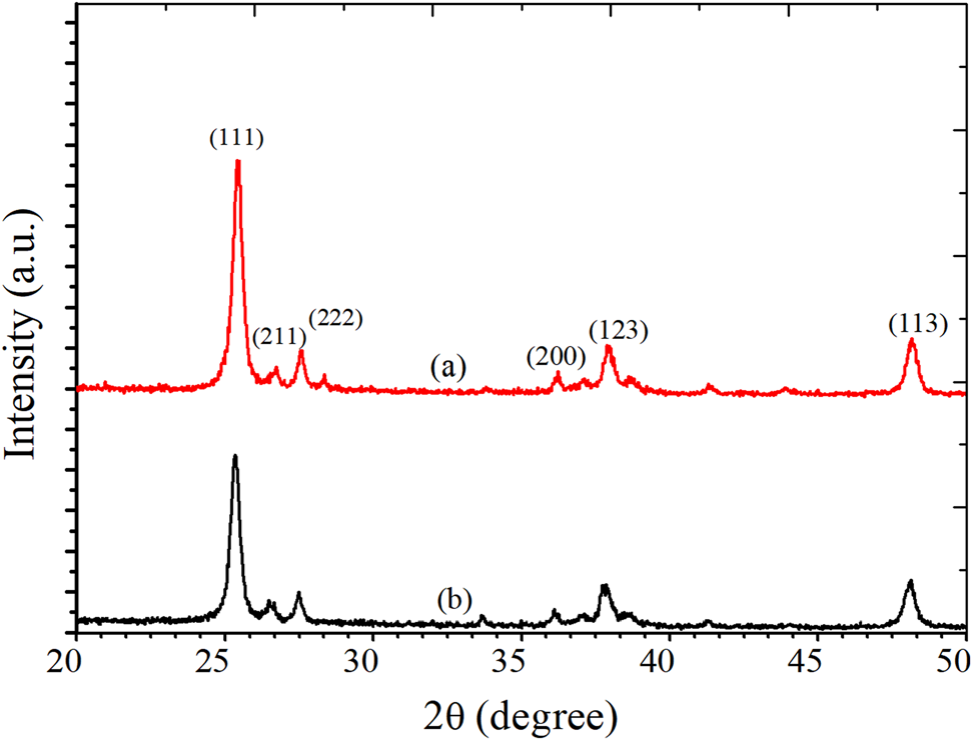
XRD patterns of (a) S_1_ and (b) S_2_ sensors.

The Scherrer formula^22,23^ was used for the calculation of the average crystallinity of the fabricated samples:1
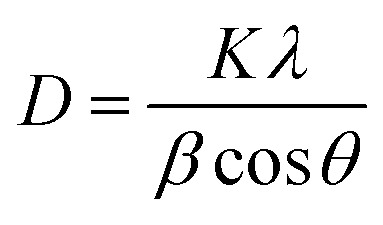
where *D* is the average size of the crystallite, *K* = 0.94 Å, *β* = full-width half maxima (FWHM) of the diffraction peak and *θ* is the angle of diffraction. The XRD pattern shows a high-intensity peak centered at 25.7°, which is assigned to the TiO_2_ plane (111). Other low-intensity peaks are assigned to the planes (211), (222), (200), (123), and (113). The XRD patterns of S_1_ (undoped-TiO_2_) and S_2_ (2 wt% CdS-TiO_2_) are shown in Fig. 2. The minimum crystallinities of TiO_2_ and 2 wt% CdS-TiO_2_ were 45.2 nm and 39.1 nm, respectively, with d spacing and FWHM of 1.775 Å and 0.1881°, respectively, indicating their nanocrystalline nature in the anatase phase. S. H. Mohamed *et al.*^14^ studied the microstructural, optical, and photocatalytic properties of CdS-doped TiO_2_ thin films. They prepared CdS doped TiO_2_ thin films on glass substrates with higher CdS contents (3, 6, 9, and 12 wt%) and showed that there are 3.35% and 5.32% reductions in crystallite size at 3 wt% and 6 wt% CdS contents, respectively. However, larger reductions in crystallite size, ∼13.49%, were found in the present investigation with smaller (2 wt%) CdS-doping in TiO_2_. The large reduction in the crystallite size played a vital role in enhancing the sensing response to test gases.

**Fig. 3:**
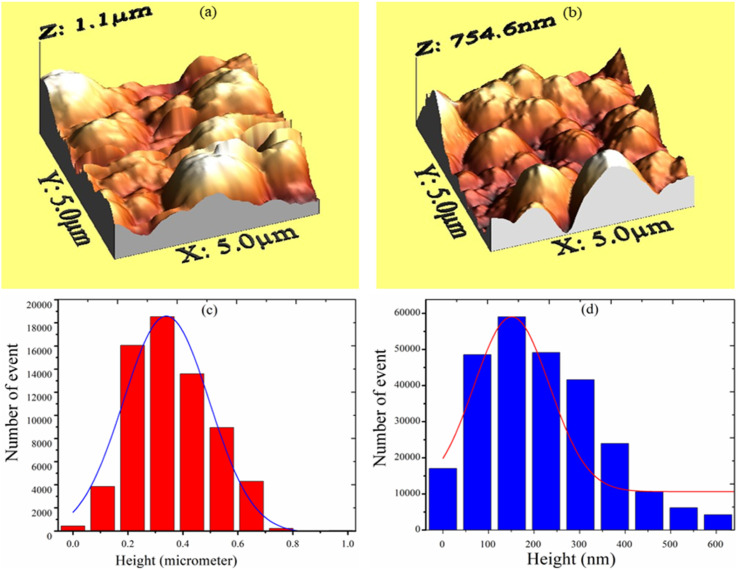
AFM 3-D structure for sensor (a) S_1_, (b) S_2_ and AFM histogram of sensor (c) S_1_, (d) S_2_.

**Fig. 4:**
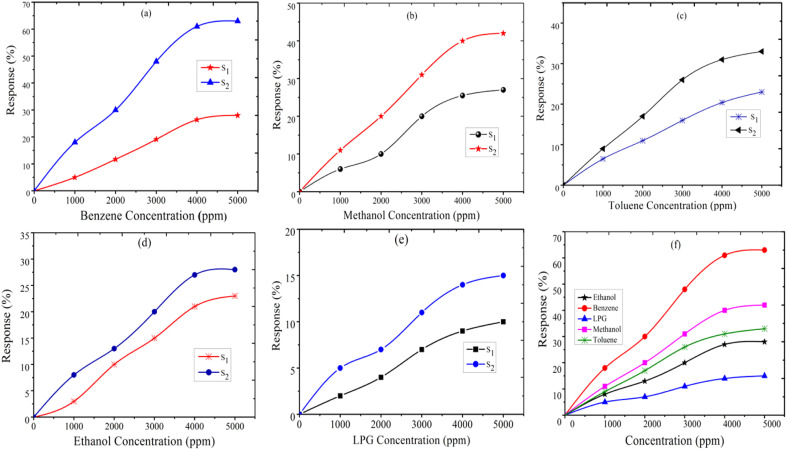
The response of sensor S_1_ and S_2_ to (a) benzene, (b) methanol, (c) toluene, (d) ethanol, (e) LPG, and (f) comparative response of sensor S_2_ to test gases.

Earlier, Vishwakarma *et al.*^24^ reported XANES (X-ray absorption near-edge spectroscopy) analysis and structural properties of CdS-doped TiO_2_ films. The result demonstrated that CdS induced a modification in the electronic structure of TiO_2_ film. It was observed that 2 wt% CdS-doping results in a phase change to the rutile phase from the anatase phase. The surface morphology of the deposited TiO_2_ film for various concentrations of CdS was investigated using AFM at room temperature in the non-contact mode. The AFM signal was recorded over an area of 250 × 250 nm^2^. The obtained data were plotted using the software WSxM.5.0 Develop.8.3. The surface morphology of the film strongly depended upon the CdS dopant concentration. The average grain size was obtained by statistically fitting the distribution curve with the Gaussian function, and it was found that the grain size reduced with CdS content (Fig. 4). The decrease in crystallite size of TiO_2_ with CdS doping was consistent with XRD measurements and confirms that the fabricated structures are polycrystalline. AFM 3D and bar diagrams for sensors S_1_ and S_2_ are presented in Fig. 3(a–d). The crystallite size, grain size, roughness, rms roughness, and surface skewness are listed in Table 1. It is observed that the surface skewness of the samples increases with CdS concentration. It is evident from Fig. 3(c and d) that grain size and roughness are reduced with CdS-doping, as listed in Table 1. The reduction in crystallite size, grain size, and roughness with 2 wt% CdS contents leads to enhancing the sensing capability of the films for various gases, including benzene, methanol, ethanol, LPG, and toluene.

**Table 1:** Crystallite size, grain size, roughness, and skewness of S_1_ and S_2_ sensors

Sensor no.	Crystallite size (nm)	Grain size (nm)	Roughness (nm)	Surface skewness
S_1_	∼45.2	∼63.2	∼23.2	−0.331
S_2_	∼39.1	∼52.4	∼16.1	0.201

### 3.2. Gas sensing behavior and mechanism

**Fig. 5:**
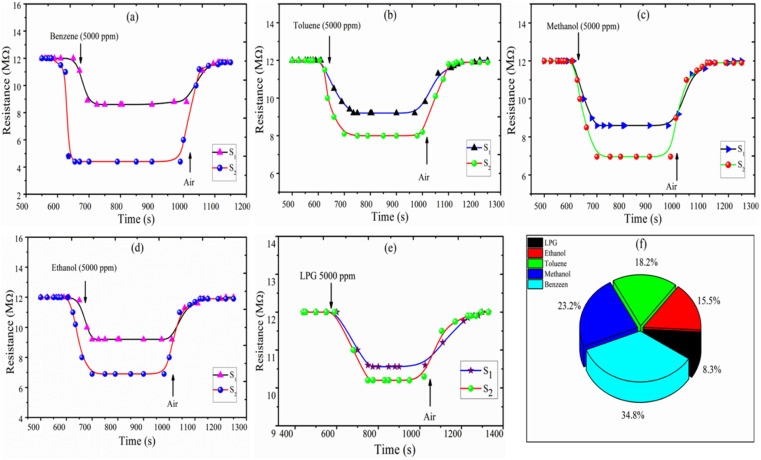
Resistance of sensors S_1_ and S_2_*versus* transient response time (s) for (a) benzene, (b) toluene, (c) methanol, (d) ethanol, (e) LPG, and (f) the selectivity of ethanol, LPG, methanol, toluene, and benzene for the S_2_ sensor.

The response of the sensor was determined using the following formula:2
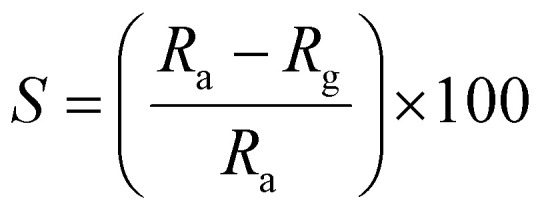
where *R*_g_ and *R*_a_ are the resistance of the sensor in the presence of gas and clean air, respectively.^25,26^ The response of sensors S_1_ and S_2_ against gas concentrations is demonstrated in Fig. 4(a–e). Fig. 4(a) represents the response of sensors S_1_ and S_2_ to benzene. It is evident from Fig. 4(a) that the response (63) of sensor S_2_ is 2.25 times higher than that of sensor S_1_. Fig. 4(b) delineated the response to methanol gas and it is 31 and 42, for sensors S_1_ and S_2_, respectively. The responses of sensors S_1_ and S_2_ to toluene, ethanol, and LPG, are presented in Fig. 4(c–e). The responses of sensor S_1_ to toluene, ethanol, and LPG were 23, 23, and 10, while those of sensor S_2_ to the same gases were 33, 28, and 15, respectively. Comparative sensor S_2_ responses to different concentrations of ethanol, benzene, LPG, methanol, and toluene at room temperature are shown in Fig. 4(f). According to the ratio of maximal sensor S_2_ responses, the response to benzene was the greatest, which was approximately 4.2 times higher than that of LPG, 2.25 times higher than that of ethanol, 1.5 times higher than that of methanol, and 1.92 times higher than that of toluene. As shown in Fig. 5(f), the sensor response measurements of S_1_ and S_2_ show that CdS doping leads to a significant improvement in the response and is more selective to benzene over other test gases.

Selectivity is one of the most important parameters of the sensor and is defined as:^21–27^3
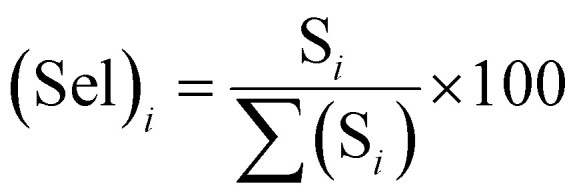
where *S*_*i*_ is the response to the test gas and Σ(*S*_*i*_) is the sum of the responses to all tested gases. Here, we calculated selectivity using eqn (4) as follows:4
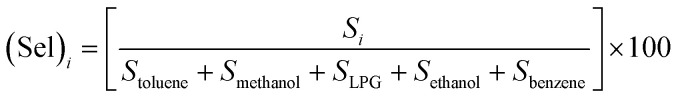


The selectivity of sensors S_1_ and S_2_ for benzene, methanol, ethanol, LPG, and toluene gases are displayed through the Pi diagram, and it is ∼34.8%, ∼23.2%, ∼15.5%, ∼8.3%, and ∼18.2%, respectively. It can be seen from Fig. 5(f) that sensor S_2_ is more selective to benzene than to other test gases.

The resistance *vs.* transient response and recovery curve of the fabricated thick film sensors S_1_ and S_2_ at a 5000 ppm concentration of the benzene, toluene, methanol, ethanol, and LPG gases, are shown in Fig. 5(a–e). The response time is evaluated by the time taken to attain 90% of the maximum response with exposure to gases and the recovery time is measured as the time taken to reach 10% of the initial value in the absence of test gases. Fig. 5(a) shows that the response time and recovery time of sensor S_2_ reduced from 65 s to 25 s and 180 s to 103 s, respectively, for benzene (5000 ppm at 300 K). The response and recovery time curves of toluene, methanol, ethanol, and LPG gases are plotted in Fig. 5(b–e). The comparative response, selectivity, response time, and recovery time of the sensors S_1_ and S_2_ for test gases are shown in Table 2. The measurement shows that the 2 wt% CdS doped TiO_2_ thick film sensor S_2_ is a suitable detector for benzene with better response and selectivity.

**Table 2:** Response, selectivity, response time, and recovery time for thick film sensors S_1_ and S_2_

Hydrocarbons	Response (%)		Selectivity (%)	Response time (s)		Recovery time (s)	
	S_1_	S_2_	S_2_	S_1_	S_2_	S_1_	S_2_
Benzene	**28**	**63**	**34.8**	**45**	**15**	**88**	**73**
Toluene	23	33	18.2	90	76	199	172
Methanol	27	42	23.2	89	72	200	155
Ethanol	23	28	15.5	96	85	199	181
LPG	10	15	8.3	95	83	210	181

A theoretical model is presented to describe the sensing behavior of the fabricated CdS-TiO_2_ thick film sensor (S_1_ and S_2_). A relation between response *S* and the concentration of gas *C* is given by.^28^5
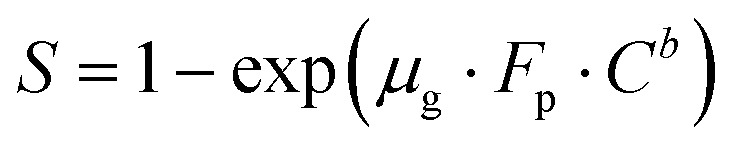
where *μ*_g_ is an arbitrary constant (A^−1^ cm^2^) whose value depends on the gas under consideration and the geometry of the sensing film, *C* is the gas-concentration in percentage (the ratio of vapor volume to chamber volume), *b* is a dimensionless constant, and *F*_P_ is the Frenkel–Poole emission constant.
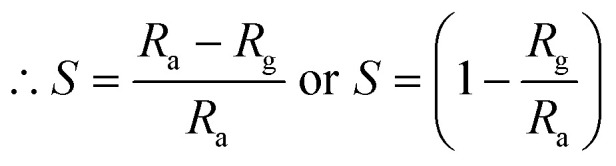


Using eqn (2) and (5) we have,
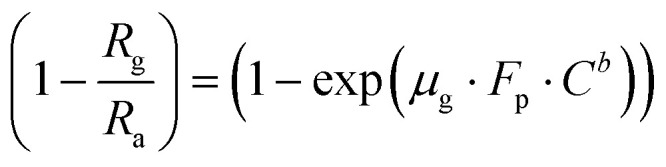
6
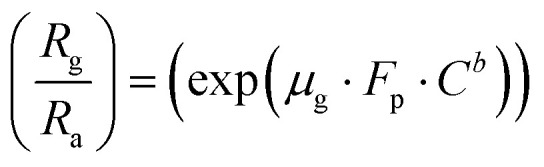


Taking the log of both sides of eqn (6)7
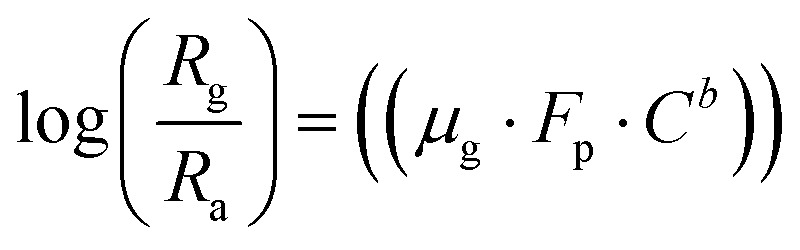


Again, taking the log of both sides eqn (7) we have,
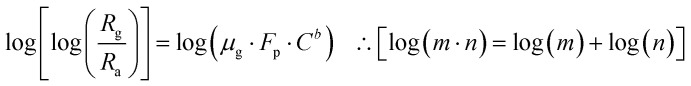
8
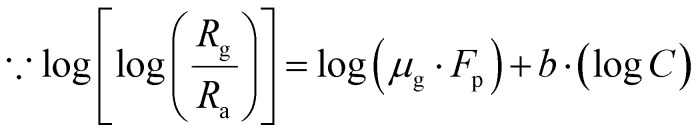


Comparing eqn (8) with *Y* = *m*·*X* + *A*.

Here slope *m* = *b* (constant) and intercept *A* = log(μ_g_·*F*_p_).

Where *F*_p_ is the Frenkel–Poole emission constant and is defined as:9
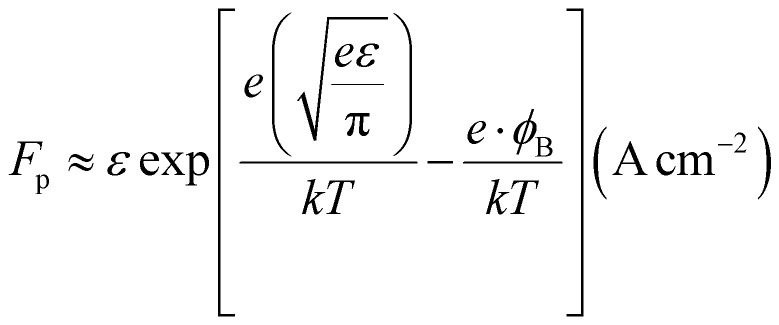
where *ε* = combined electric field (V cm^−1^), *e* = electronic charge, *φ*_B_ = barrier height, *k* = Boltzmann constant, and *T* = absolute temperature. We know that the relation between voltage and the electric field is, *ε* = *V*/*d*. Thus, eqn (9) can be written as:
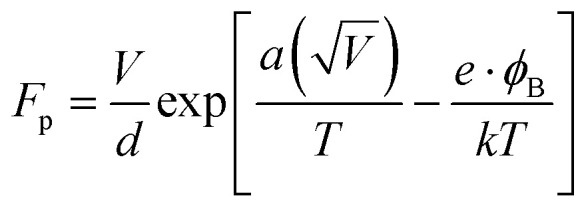
where 
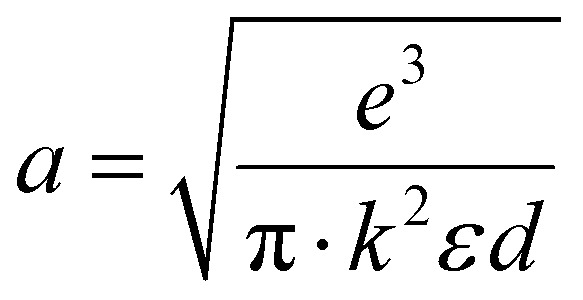
 and is a constant.^28^

**Fig. 6:**
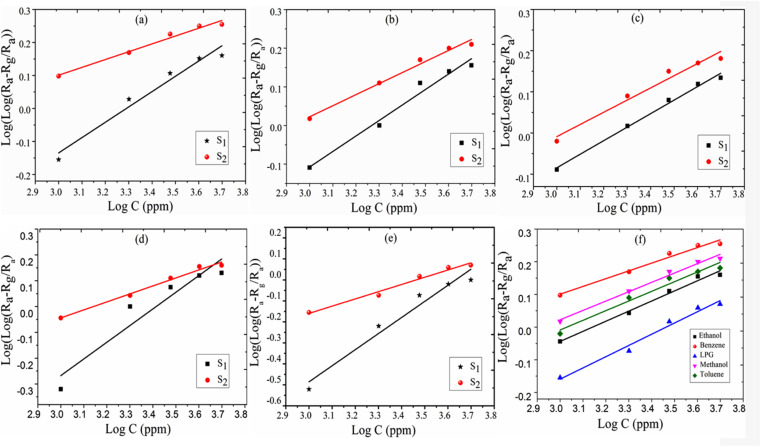
Logarithmic presentation of sensing response *vs.* logarithmic presentation of test gas concentration (ppm) (a) benzene, (b) methanol, (c) toluene, (d) ethanol, (e) LPG; and (f) the comparative response for sensor S_2_.

The logarithmic sensing response as a function of all-test gas concentrations, log *C*, is plotted in Fig. 6(a–e) portraying a linear logarithmic response (*R*^2^ = 0.9767) with benzene concentration. The Frenkel–Poole emission constant parameter is found as *μ*_g_·*F*_p_ ≈ (−0.630 ± 0.068) while the constant parameter *b* = (0.242 ± 0.019). From Fig. 6(a), it is observed that the response increases more linearly up to 4000 ppm and then tends to attain stagnation beyond a concentration of 5000 ppm. The obtained experimental results fit with the proposed model and log(*C*) variation with log(*R*_a_ − *R*_g_/*R*_a_), producing a straight line. The comparative logarithmic concentration for the S_2_ sensor is shown in Fig. 6(f).

The response behavior of the fabricated sensors to the exposed hydrocarbon gases has also been explained based on adsorbed oxygen species and lower crystallite size of TiO_2_ with CdS-doping. One of the important factors in controlling the response of a chemo-resistive metal oxide gas sensor is particle size (*D*).^29,30^ Enhancement in the response of the fabricated sensor can be achieved through modulation of microstructural properties, specifically the grain size. Smaller grain sizes tend to yield superior gas sensing responses. However, it is crucial to strike a balance to ensure that the structural stability of the sensor is maintained. The response of a thick film sensor based on TiO_2_ can be correlated with the characteristics of the space charge layer (*L*)^31,32^ and it is reported that for *D* < 2*L*, the depletion layer does not restrict just at the surface but extends throughout the grain. The reduction in particle size with 2 wt% CdS-doping increases the alive surface area of the fabricated thick film sensor, which results in higher adsorption of oxygen species and increased response of sensors to the reducing gases.^33,34^ A comparison table of sensing response, operating temperature, and response time for hydrocarbons is listed in Table 3.

**Table 3:** Comparison table of sensing response, operating temperature, and response time for alcoholic and acetone gas

Sample	Text gas	Operating temperature (°C)	Sensitivity (%)	Response time (s)	References
CdS-TiO_2_	Acetone	—	71	55	21
CdS-TiO_2_	Propanol	—	63	62	27
CdS-SnO_2_	Propanol	200	78	19	35
CdS-SnO_2_	Toluene	200	51	—	36
PbO-SnO_2_	Ethanol	200	88	—	37
Sb_2_O_3_-SnO_2_	Ethanol	150	66	19	38
ZnO/TiO_2_	Propanol	—	23	10	39
SnO_2_ nanorods	Iso-propanol	325	11.2	6	40
SnO_2_-Pd-Pt-In_2_O_3_	Methanol	160	320.7	32	41
Ce-doped SnO_2_	Acetone	270	50.5	—	42
CdS-TiO_2_	Benzene	27	63	15	Present work

When exceeding TiO_2_ bandgap energy, the electron is promoted from the valence band to the conduction band TiO_2_. The sensing mechanism of the fabricated sensors (S_1_, S_2_) can be determined as the resistance of materials varies with the oxygen molecules absorbed at the surface and the concentration of reducing gas species. When metal oxide comes in contact with air, oxygen molecules from the ambient air are adsorbed onto the surface of the metal oxide. At the grain boundaries electrons are trapped and a barrier is built around each grain. The adsorbed oxygen molecules are subsequently converted to ions after capturing an electron from the conduction band.^35^Eqn (10)–(13) are possible representations for the adsorption of the oxygen species (a, stands for air, ad stands for adsorption, and g stands for gas).^43,44^

10 O_2(a)_ → O_2(ads.)_

11 O_2(a)_ + e^−^ → O_2(ads.)_^−^

12 O_2(ads.)_^−^ + e^−^ → 2O^−^

13 2O^−^ + e^−^ → O_(ads.)_^2−^

**Fig. 7:**
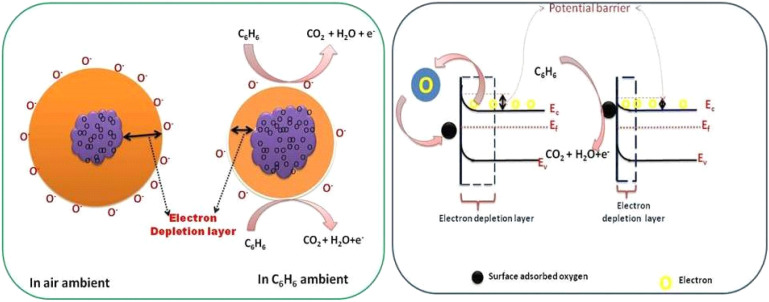
Role of oxygen species and effect of CdS on the TiO_2_ band structure for benzene gas detection.

TiO_2_ is an n-type semiconductor material with an electron as a majority carrier. When C_6_H_6_ molecules get in contact with the TiO_2_ surface, adsorbed oxygen species readily interacts with hydrocarbons by releasing electrons back to the conduction band of TiO_2_. Thereby, resistance decreases because of the electron-donating nature of C_6_H_6_ and subsequently the conductivity increases. The measurement reveals that the resistance of sensors S_1_ and S_2_ decreases with exposure to various test gases, namely, benzene, toluene, ethanol, methanol, and LPG in ambient air. The decrease in resistance of the sensor in the presence of benzene and other test gases may be due to decrease in the size of the depletion layer, as shown in the band diagram of CdS-TiO_2_ thick film (Fig. 7). The understanding of the reaction mechanism on the CdS-TiO_2_ surface in the presence of VOCs is represented as,

14 C_*x*_H_*y*_ + *n*O^*m*−^ → *x*CO_2_ + H_2_O + *m*·*n*e^−^

where *m* and *n* are integers. In the case of benzene *x* = 6, and if *m* = 1 or *m* = 2, then the possible reaction may be defined as eqn (15)–(19),

15 C_6_H_6_ + 15O^−^ → 6CO_2_ + 3H_2_O + 15e^−^

Or

16 C_6_H_6_ + 15O^2−^ → 6CO_2_ + 3H_2_O + 30e^−^

Or

17 2C_6_H_6_ + 15O_2_^−^ → 12CO_2_ + 6H_2_O + 15e^−^

Or

18 C_6_H_6_ + 12O^−^ → 6CO_2_ + 3H_2_ + 12e^−^

Or

19 C_6_H_6_ + 12O^2−^ → 6CO_2_ + 3H_2_ + 24e^−^

The possible reaction for ethanol is given in eqn (20)–(22)

20 C_2_H_5_OH + 6O^−^ → 2CO_2_ + 3H_2_O + 6e^−^

Or

21 C_2_H_5_OH + 6O^2−^ → 2CO_2_ + 3H_2_O + 12e^−^

Or

22 C_2_H_5_OH + 3O_2_^−^ → 2CO_2_ + 3H_2_O + 3e^−^

The possible reaction for toluene is given in eqn (23) and (24)

23 C_6_H_5_OH + 14O^−^ → 6CO_2_ + 3H_2_O + 14e^−^

Or

24 C_6_H_5_OH + 14O^2−^ → 6 CO_2_ + 3H_2_O + 28e^−^

The response of the fabricated sensors S_1_ and S_2_ towards benzene and other test hydrogen-containing gases can be explained based on eqn (14)–(24). When sensor S_2_ is exposed to hydrogen-containing gases, such as benzene, toluene, methanol, ethanol, and LPG, the exposed gases react with oxygen anions present on the surface and may result from H atoms, some of which cross the interface of CdS/TiO_2_. O. W. Johnson^45^ already reported the diffusion of H_2_ into TiO_2_; these hydrogen atoms are ionized to produce conduction electrons and interstitial protons. During this reaction, some carriers may get trapped inside the TiO_2_. Thus, it may be possible that there would be an increase in electrical conductivity with varying concentrations (0–5000 ppm) due to the high polarizability of TiO_2_ lattice and increase in trap charges at the CdS/TiO_2_ interface upon exposure to benzene. The faster response and recovery time of the sensor S_2_ to the test gases is attributed to a change in barrier height at grain boundaries, which is due to exposure of test gases and their interaction on the surface of TiO_2_–CdS, modulating the surface charge densities, which depend on temperature and gas exposure. Future work can be done regarding oxidation of target gases at room temperature in dry and wet conditions and its reaction with chemisorbed oxygen species.

### 3.3. Effect of relative humidity on sensing properties

**Fig. 8:**
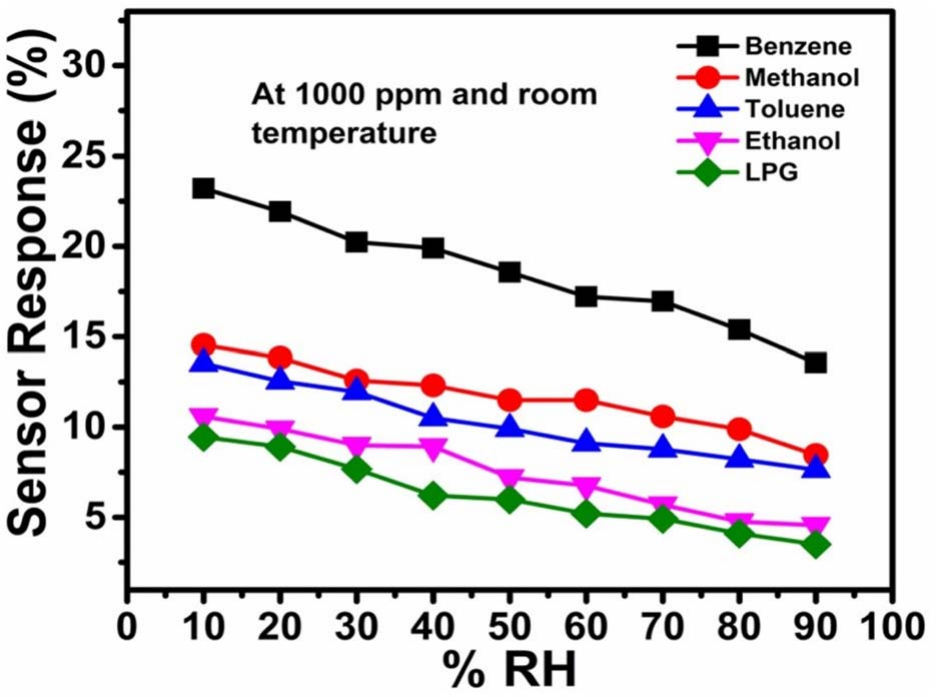
Sensor responses to various analytes at different relative humidity levels.

The effect of relative humidity on gas sensor performance is a crucial aspect for sensor development, especially when aiming for operability at room temperature. Understanding how humidity levels affect gas response properties can lead to more accurate and reliable sensors. The performance of a CdS-TiO_2_ chemo-resistive sensor at various relative humidity (RH) values for various gases is shown in detail in Fig. 8. It is clear from a detailed examination of Fig. 8 that for the majority of the studied gases, the effect of RH on sensor performance is constant; greater humidity levels are detrimental to sensor reliability in detecting the target gases. It is important to note that humidity levels at the higher end of the spectrum, between 70% and 90% RH, have the most significant impacts on the sensor response. Comparing the responses of the sensor to other gases under the same RH conditions, interesting patterns emerge. For instance, the sensor has the best response to benzene (23.21%) and the worst response to LPG (9.45%) at 10% RH. This shows that the sensor is most sensitive to benzene and least sensitive to LPG at low humidity levels. When using this sensor for applications that demand selective gas detection, these differences in sensitivity should be considered. The data also suggest RH levels at which there are considerable declines in sensor responsiveness. The sensor response to methanol, for instance, is 12.59% at 30% RH and 12.31% at 40% RH. This indicates that the sensitivity of the sensor towards methanol noticeably decreases between 30% and 40% RH. Finding these thresholds is essential for improving the performance of sensors in real-world situations with changing humidity levels. This sensor has some humidity sensitivity in terms of its anti-humidity capabilities. Despite changes in humidity levels, the ideal anti-humidity sensor would continue to respond consistently. However, this information shows that when RH rises, the performance of the sensor tends to deteriorate. Because of this, it might not be the ideal option for applications in high-humidity situations without extra moisture mitigation methods.

## 4. Conclusions

Under ambient room temperature conditions, the detecting capacities of two thick film sensors, S_1_ (undoped) and S_2_ (containing 2 wt% CdS-TiO_2_), were investigated. The test gases included benzene, toluene, methanol, ethanol, and LPG. The sample with 2 wt% CdS combined with TiO_2_ (S_2_) had the most notable response, with a sensitivity of 63% for benzene gas. Measurements reveal that the sensor is more selective to benzene than to the other test gases; its selectivity lies at 34.8%, which is much higher than that of others (table). The higher response to exposure to benzene is due to a decrease in crystallinity with the increase in CdS contents and surface modifications of the CdS/TiO_2_ structure. The addition of 2 wt% CdS in the TiO_2_ sample was found to be effective in promoting the response and selectivity. Thus, we conclude that 2 wt% CdS-TiO_2_ samples are good candidates for the growth of low-cost high-performance benzene (C_6_H_6_) gas sensors at room temperature and low humidity levels.

## Data availability

Data are available on request.

## Conflicts of interest

There are no conflicts to declare.
